# How Can We Decrease Early Dialysis Initiation? An Interactive Quality Improvement Teaching Case for Health Care Providers and Narrative Review of Quality Improvement Methodology

**DOI:** 10.1177/20543581251323947

**Published:** 2025-05-22

**Authors:** Khaled Lotfy, Epsita Shome-Vasanthan, Samuel A. Silver, Tamara Glavinovic

**Affiliations:** 1 Division of Nephrology, Department of Medicine, London Health Sciences Center, Western University, ON, Canada; 2 Division of Nephrology, Department of Medicine, University of Alberta, Edmonton, Canada; 3 Division of Nephrology, Department of Medicine, Kingston Health Sciences Centre, Queen’s University, ON, Canada; 4 Division of Nephrology, Department of Medicine, The Ottawa Hospital, University of Ottawa, ON, Canada

**Keywords:** quality improvement, quality assurance, dialysis start, education, advanced chronic kidney disease

## Abstract

**Purpose of Review:**

Quality improvement (QI) initiatives use a team-based approach to problem-solving clinical and health system issues. All QI initiatives require the coordinated efforts of health care professionals and other stakeholders to encourage the provision of evidence-based clinical care. Most clinicians understand the principles of QI but may lack the training necessary to undertake individual projects.

**Methods:**

An educational, nephrology-oriented clinical case was created based on the IDEAL study on timing of dialysis initiation, a prioritized quality indicator in several provinces. The case illustrates how to utilize commonly employed QI methodology and to provide a pragmatic framework for both developing and running a QI project. Core concepts addressed in this review include how to perform a QI chart audit, identification of a quality-of-care problem, engaging stakeholders, and how to conduct a root cause analysis that leads to selection of QI measures and change solutions. Last, plan-do-study-act (PDSA) cycles and interpretation of data using run charts are highlighted.

**Sources of Information:**

PubMed and Google scholar were used as sources of published QI methodology.

**Key Findings:**

This nephrology-oriented QI case highlights how a core set of QI principles and tools can be used to improve clinical care. This review demonstrates that determining clear goals, utilizing evidence-based guidance to improve timing of dialysis initiation, engaging the appropriate stakeholders, identifying a feasible and measurable change, and tracking if that change leads to improvement are essential components of all QI initiatives. The above framework can be utilized in a variety of clinical areas both within and beyond nephrology-specific care.

**Limitations:**

Considerations regarding QI-specific data analysis were not addressed as they were beyond the scope of this review.

## Abrégé

**Motif de la revue:**

Les initiatives d’amélioration de la qualité (AQ) misent sur une approche axée sur le travail d’équipe pour résoudre les problèmes cliniques et aborder les enjeux du système de santé. Pour favoriser la prestation de soins cliniques fondés sur des données probantes, toutes les initiatives d’AQ nécessitent les efforts coordonnés des professionnels de la santé et des parties prenantes. La majorité du personnel clinique comprend les principes de l’AQ, mais certains individus pourraient ne pas avoir reçu la formation nécessaire pour entreprendre des projets individuels.

**Méthodologie:**

Un cas clinique éducatif, axé sur la néphrologie, a été créé à partir de l’étude IDEAL qui porte sur un indicateur de qualité prioritaire dans plusieurs provinces: l’amorce de la dialyse. Ce cas démontre comment utiliser la méthodologie d’AQ couramment employée et fournir un cadre pragmatique pour le développement et la réalisation d’un projet d’AQ. Parmi les principaux concepts abordés dans cette revue figurent la réalisation d’un audit des dossiers d’AQ, l’identification d’un problème de qualité des soins, la mobilisation des parties prenantes et l’analyse des causes profondes qui mènent à la sélection de mesures d’AQ et de solutions de changement. Les cycles PDSA (Plan-Do-Study-Act) et l’interprétation des données à l’aide de graphiques de séquences sont également soulignés.

**Sources:**

PubMed et Google Scholar ont été utilisées comme sources pour la méthodologie d’AQ publiée.

**Principaux résultats:**

Ce cas axé sur la néphrologie montre comment l’utilisation d’un ensemble de principes et d’outils d’AQ peut contribuer à améliorer les soins cliniques. Cette étude démontre que toute initiative d’AQ réussie passe par certains éléments essentiels: la définition d’objectifs clairs, l’adoption de directives fondées sur les données probantes pour l’amorce de la dialyse, la mobilisation des parties prenantes concernées, l’identification d’un changement réalisable et mesurable, et le suivi de l’amélioration apportée par ce changement. Ce cadre peut être employé dans plusieurs domaines cliniques à la fois, pour les soins de néphrologie et au-delà.

**Limites:**

Les éléments relatifs à l’analyse des données propres à l’AQ n’ont pas été abordés, car ils dépassaient la portée de la présente revue.

## Introduction

Quality improvement (QI) initiatives use a team-based approach to problem-solving clinical and health system issues and require knowledge of the best available clinical evidence, but also knowledge of how best to prioritize ideas and track the changes that are tested along the way. All QI initiatives require coordination between health care professionals and other stakeholders. These projects should align with patients’ and health care professionals’ priorities, and determining how to organize, document, and plan each step is critical for successful and sustainable change. As the majority of Canadian nephrologists who participate in or lead QI initiatives may not have formal training in this area,^1^ the purpose of this educational, nephrology-oriented clinical case and review of QI methodology is to provide a framework for clinicians to develop a QI project, to highlight core QI concepts, and to guide clinicians in successfully implementing and troubleshooting a QI project.

## Methods

A PubMed and Google Scholar search was used to include relevant QI methodology. We then created a fictional clinical case to illustrate how commonly employed QI tools can be used to improve clinical care at a local level.

## Review

### The Clinical Case

A 70-year-old woman is followed in your local multidisciplinary kidney clinic. She feels well and has no symptoms suggestive of uremia. Her clinical examination shows mild ankle edema and metabolic parameters are normal. Her eGFR is 11 mL/min/1.73 m^2^ from 12 mL/min/1.73 m^2^ the month prior. She asks the team, “. . . when should I start dialysis?”

**Table:** 

***Question 1***: *Based on available evidence, when should we initiate dialysis?* *a) Start now, do not wait until she has a clinical or metabolic indication* *b) Defer dialysis until there is an indication (eg, hyperkalemia, volume overload, symptoms suggestive of uremia) or eGFR drops to 6 mL/min/1.73 m^2^*, *whichever comes first.* *c) Uncertain*

*(see the following text for answers)*

The nephrology team ask themselves, “What does the evidence tell us about when to start dialysis?” and “What QI strategies will ensure we follow evidence-based care?”

### eGFR and Dialysis Initiation

The suggested timing of dialysis initiation comes from the IDEAL Study, a multicenter randomized control trial that studied patients with advanced chronic kidney disease (category G5) randomized to early dialysis initiation (eGFR, 10-14 mL/min/1.73 m^2^) or late (eGFR 5-7 mL/min/1.73 m^2^).^2^ Over 3.6 years of follow-up, there was no difference in survival or other clinical outcomes between the 2 groups. The conclusion from the IDEAL study was that the intent-to-defer dialysis strategy does not negatively impact clinical outcomes including survival and likely results in substantial cost savings, due to higher dialysis and transportation costs in the early-start group. A limitation of this study was their use of the Cockcroft-Gault equation rather than MDRD. The assay used to develop the Cockcroft-Gault was likely 10% to 20% higher than MDRD, and at the time of dialysis initiation, the eGFR using the Cockroft-Gault formula versus the MDRD formula was 12 versus 9.8 and 9 versus 7.2 mL/min/1.73 m^2^ for the early versus late start groups, respectively.^3^ An intent-to-defer strategy is accepted by the Canadian Society of Nephrology (CSN), who released clinical practice guidelines on this topic in 2014^4^; therefore, the answer to the question is b.

Patient engagement in developing QI initiatives is integral to any project, as priorities may differ between those of health care providers, including dialysis-free time and the ability to travel versus lab values.^5^ A model of shared decision-making between the patient and health care team should focus on addressing the various priorities, and discussion and decisions surrounding kidney care should focus on the expected changes of advancing kidney disease.^6,7^

### Approaching eGFR and Dialysis Initiation From a Quality Improvement Perspective

The first steps in conducting a QI project involve understanding local program practice. Reviewing local data may reveal unanticipated findings. For example, between 2001 and 2007, the mean estimated glomerular filtration rate at initiation of dialysis in Canada increased from 9.3 to 10.2 mL/min per 1.73 m^2^, and the proportion of early starts rose from 28% to 36%.^8^

#### Chart Audit—how many charts should be reviewed in your kidney clinic to identify this quality-of-care problem?

A common misperception among care providers is that we need an extremely large sample size to obtain an answer.

**Table:** 

***Question 2***: *If it is expected that 50% of patients in your clinic are starting dialysis with an eGFR >9.5 mL/min/1.7 3m^2^*, *and your desired performance is 30%, how many charts must you review to be convinced that a quality-of-care problem exists?* *a) 250* *b) 100* *c) 40* *d) 12*

In Table 1, Etchells et al demonstrate the approximate sample size required to reject the null hypothesis (using a conventional 2-tailed *P* value of .05) that observed performance (from an audited sample) is consistent with the desired system performance. This information can help determine how many charts require auditing. Clinicians may be surprised to see how few charts need to be audited to determine a quality-of-care problem.

**Table 1.** Minimum Sample Sizes Required for QI Projects Based on Observed and Desired System Performance.

Observed system performance (%)	Desired system performance	
	80%	90%
**95**	26	140
**90**	70	Not applicable
**85**	260	180
**80**	Not applicable	50
**75**	280	28
**70**	80	20
**66**	45	15
**60**	25	10
**50**	12	6
**40**	10	5
**20**	5	5

*Source.* Adapted from Etchells et al.

*(Answer to question 2 is d)*

#### When are patients starting dialysis in your practice?

The team reviews eGFR at dialysis initiation in the kidney clinic over the preceding 6 months, and they find that 50% of patients start dialysis with an eGFR of >9.5 mL/min/1.73 m^2^. A literature review of this topic can also be used to determine baseline performance and root causes.

#### Creating a Problem and Aim Statement

Having identified a quality-of-care problem, the next step is to develop a “problem statement.” A problem statement is a framework upon which we then create an “aim statement”—the focus of our quality improvement initiative.

**Table:** 

**Example of a problem statement:** *Patients are often started on dialysis with an eGFR>9.5 mL/min/1.73 m^2^*. *This practice does not improve patient outcomes.*

Effective aim statements are **S.M.A.R.T**: They are *specific* about what they target to achieve. They are *measurable* and should include a specific outcome or process measure (eg, no. of patients doing *x*). They should be *achievable*, where the desired level of improvement should be feasible. Determining the level of improvement that is feasible is often challenging and may be a moving target. This depends on many factors, including the resources available to establish QI interventions. They should be *relevant*, where the aim should be meaningful to stakeholders. Last, the aim statement should be *time-bound*, where a realistic target date should be set.

**Table:** 

***Question 3. Based on the clinical case and your literature review***, *choose the most appropriate aim statement for this project*: *a) We aim to decrease the proportion of patients starting dialysis early.* *b) We aim to decrease the proportion of patients starting dialysis early* *c) (eGFR >9.5 mL/min/1.73 m^2^).* *d) We aim to decrease the proportion of patients starting dialysis early* *e) (eGFR >9.5 mL/min/1.73 m^2^) to <30% by the end of the next calendar year.*

*The answer to question 3 is c (a is incorrect because it is neither specific nor time bound. b is incorrect because it is not time bound. c is correct—it defines an early start, provides a target and a date. It is achievable, as some programs are below 20%)*

The next steps are to create a *root cause analysis team*. This includes stakeholders invested in the outcome with knowledge of the issue. Creating a root cause analysis team is critical. Those affected by the proposed changes should be those helping create and guide the process.

#### Who are our stakeholders?

Stakeholders in this QI initiative can include, for example, the nephrology division head, nephrologists, nurse practitioners, nursing staff, dieticians, social workers, clinical managers, nephrology trainees, patients, caregivers, and information technology support staff.

**Figure 1.**
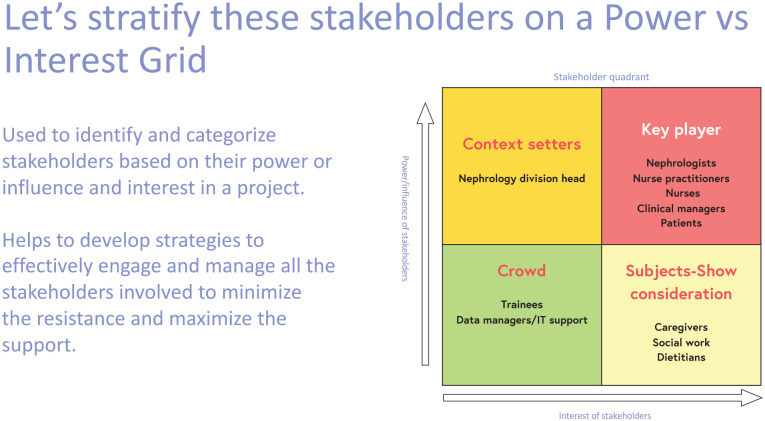
Power vs interest grid.

To help identify and categorize stakeholders based on their influence and interest in a project, a *power vs interest grid* can be an effective visual tool (Figure 1).^9,10^ This grid can help develop strategies to engage and manage all involved stakeholders, to minimize resistance against the project, and to maximize support.

### Patient Engagement

Engaging a patient champion while choosing the members of your team is an essential way of incorporating the voices of patients and caregivers into decisions affecting clinical care. Patient engagement improves communication between patients and health care providers, leading to increased patient and provider satisfaction, which may lead to a better embrace of potential changes. This empowers patients to become involved in their own health care and can result in meaningful changes to health care services. Any potential barriers to patient engagement within an organization need to be addressed.^11^

**Table:** 

***Question 4.*** *Which one of the following is not a root cause analysis tool?* *a) The 5 Why’s* *b) Pareto chart* *c) Fishbone / Ishikawa diagram* *d) Driver diagram*

*The answer to question 4 is d. The 5 why’s, Pareto chart, and fishbone / Ishikawa diagram are all root cause analysis tools. A driver diagram is a tool designed to help prioritize change ideas.*

Identifying a cause before developing potential solutions is a key tenet of QI work. To figure out why patients may be starting dialysis at higher eGFRs, the QI team may use several root cause analysis tools including the 5 Why’s, a fishbone / Ishikawa diagram, a failure mode and effect analysis, and a Pareto diagram. Patient champions will play a big part in identifying potential areas for improvement. This step may include consulting with the patient champions who are part of your team or reaching out to a larger number of patients through surveys, interviews, or focus groups.

**Figure 2.**
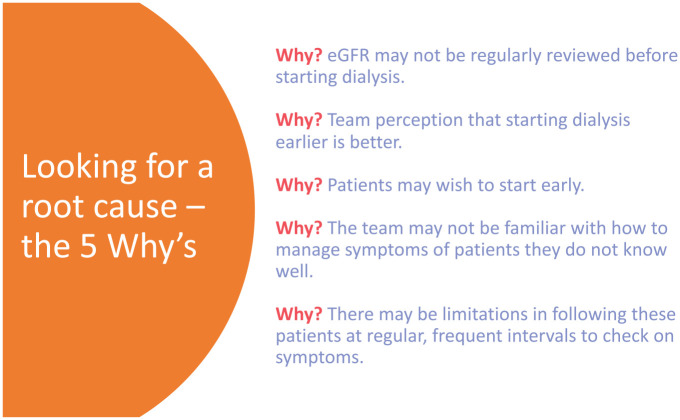
The 5 Why’s—Determining the root cause of a clinical problem.

**Figure 3.**
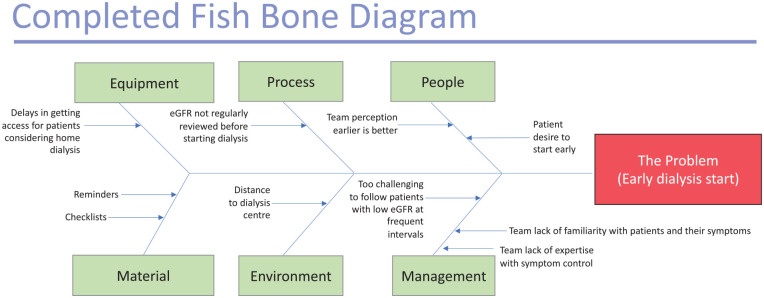
Example of a Fishbone / Ishikawa diagram considering the reasons why people followed in the multidisciplinary kidney clinic may be starting on dialysis early.

1. *The 5 Why’s:* a brainstorming exercise where team members consider the root cause behind patients starting dialysis at higher eGFRs (Figure 2).^12^

2. *Fishbone* ***/*** *Ishikawa diagram:* A fishbone diagram (Figure 3) is a cause-and-effect diagram with 6 process categories. This diagram helps identify possible causes of a problem and helps brainstorm causes relating to specific categories. Each “fishbone” branch is labeled with a category. The 4Ss, 5Ps, 6Ms is an example of different categories that can be used (*4S: surroundings, suppliers, systems, skills; 5P: patients, providers, policies, procedures/processes, place/equipment; 6M*: ***m*** *achine*, ***m*** *ethod*, ***m*** *aterials*, ***m*** *easurement, hu(* ***m*** *an), and environment (* ***m*** *other nature).*

3. *Failure mode and effect analysis (FMEA):* This tool helps evaluate processes to identify how and where they might fail and to assess the impact of various failures. The FMEA tool helps us to proactively change processes to prevent future failures instead of reacting once failures have occurred (see Table 2).^12^

**Table:** 

***Question 5.*** *What is the Pareto principle?* *a) That only 50% of root causes should be prioritized* *b) That root causes must be identified to solve a QI problem* *c) That 80% of outcomes come from ~ 20% of the causes*

**Table 2.** Example of Failure Mode and Effect Analysis (FMEA) Tool.

Steps	Failure mode	Failure causes	Failure effects	Likelihood of occurrence (1-10)	Likelihood of detection (1-10)	Severity (1-10)	Risk profile number (RPN)	Actions to reduce occurrence of failure
	What could go wrong?	Why would the failure happen?	What would be the consequences of the failure?	10 being the most likely	10 being the most likely NOT to be detected	What is the likelihood that the failure mode will cause severe harm?	Multiply the 3 scores. The lowest score will be 1, the highest possible score will be 1000.	List the possible actions to improve safety systems, especially for failure modes with the highest RPNs.
**1**	Patient symptom checklist completed.	Lack of time by allied health.	Patient may be experiencing symptoms that are not picked up by the team.	5	7	7	245	Reminder in template patient note to address symptom checklist.
**Etc.**								

*The answer to question 5 is c—the Pareto principle states that 80% of the outcomes or consequences can be traced back to roughly 20% of the causes. This is otherwise known as the 80/20 rule.*

**Figure 4.**
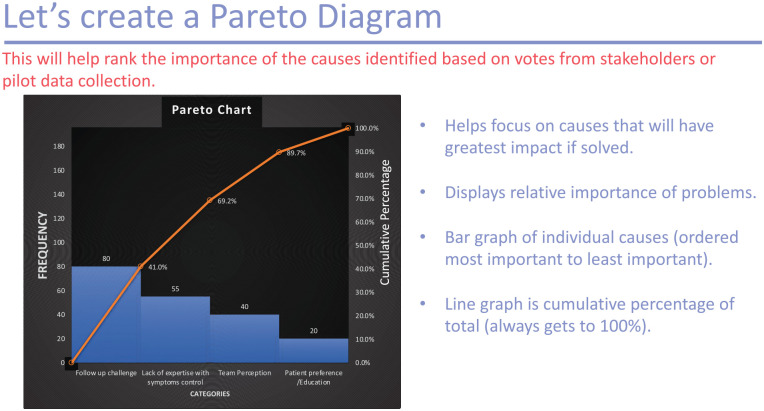
Example of a Pareto diagram.

4. *The Pareto diagram:* A Pareto diagram is a bar chart depicting how often different categories of events occur. The most commonly occurring event may be the root cause and this helps maintain focus on causes that will have the greatest impact if solved (Figure 4).^12^

**Figure 5.**
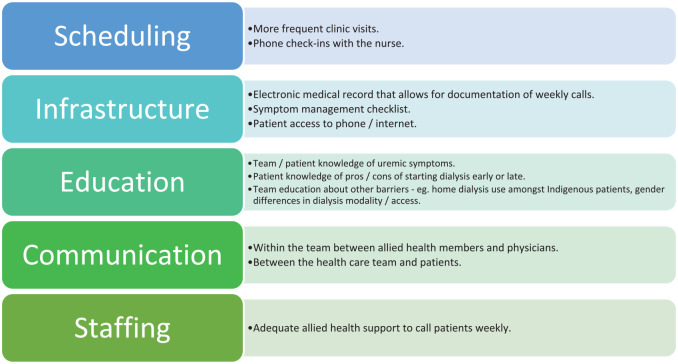
Example of an affinity diagram where change ideas are developed and categorized.

Based on the root cause analysis, the QI team decide the greatest potential impact will stem from addressing

- Difficulties reviewing patients with low eGFR regularly in clinic
- A lack of expertise with symptom management

#### Next steps: brainstorming solutions and change ideas

Several tools have been developed to encourage brainstorming and organizing change ideas that address the root causes identified above. An affinity diagram (Table 3) allows teams to be creative but also to include and categorize change ideas that are both measurable and achievable. These change ideas should be directed at solving the root causes (eg, difficulty reviewing patients with low eGFR on a regular basis in the clinic and lack of expertise with symptom management).

**Table 3.** Hierarchy of effectiveness.

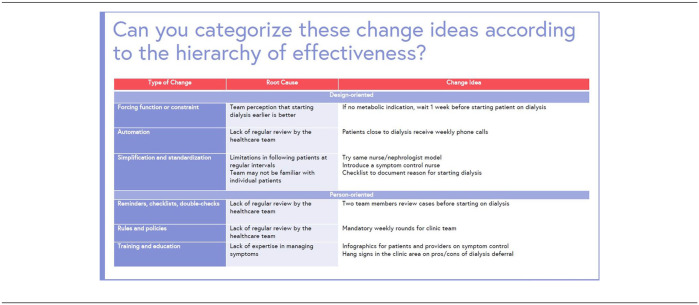

A visual representation of a hierarchy of effectiveness is another brainstorming tool that helps organize the following change ideas by increasing effectiveness (see Table 4).^13^

**Table 4.** Example of different QI measures and their definitions.

QI Measure	Definition	Examples based on Clinical Case
**Outcome measure**	- A measure of what we are trying to achieve by capturing system performance. - Usually you have only one.	- The proportion of patients starting dialysis with an eGFR <9.5 mL/min/1.73 m^2^.
**Process measure**	- Measures the things you are doing to achieve your aim capturing the changes your QI efforts make to the inputs or steps that contribute to system outcomes. - These can be different for each PDSA cycle. - There are often multiple process measures.	- The proportion of patients receiving weekly telephone calls to evaluate uremic symptoms. - The proportion of visits where a symptom management checklist is completed.
**Balancing measures**	- Evaluate system performance and potential changes. - Measures unintended consequences or trade-offs.	- The additional time spent by nursing staff per patient per week. - Additional time spent calling patients to review symptoms.

**Table:** 

***Question 6.*** *Which type of change idea may be most effective based on the hierarchy of effectiveness that will help reduce the proportion of patients starting dialysis early?* *a) Forcing function and constraints (eg, if no metabolic indication, wait 1 week before* *b) starting the patient on dialysis)* *c) Training and education (eg, infographics for patients and providers on symptom control)* *d) Simplification and standardization (eg, introduce a symptom control nurse)*

*The answer to question 6 is a—design-oriented features are sometimes the most effective at creating change as they rely less on human factors and will create less variability between health care providers.*

#### How do we decide which change idea to test first?

A “Possible / Implement / Challenge / Kill” (PICK) chart helps categorize and identify whether a change idea will be easy or difficult to implement.^14^ Items in the left upper corner will have a high payoff and are relatively easy to implement. Items in the bottom right corner will have a lower payoff—they may be challenging to implement, and potentially reasonable to defer. Remember that change ideas should address root causes no matter where they fall on the PICK chart.

Once a change idea has been selected for further testing, it is important to track whether our changes will lead to improvement. The Institute for Healthcare Improvement (IHI) Model for Improvement is a model which focuses on setting aims and developing measures to see whether a change results in improvement. At the core of this model is the Plan-Do-Study-Act cycle, where small scale interventions are implemented to test for change, and where subsequent cycles are modified to learn from these tests.^15,16^ In the “Plan” phase, we describe the objective and the change being tested. Predictions of what will happen are formulated, and action steps are created, with a plan for collecting data. The “Do” step is where the change ideas are tested, data are collected, and we describe what happens. We then “Study” and analyze the collected data, compare outcomes with what was originally predicted, and summarize what has been learned. Last, we “Act” and decide the next best steps, considering any changes for the next Plan-Do-Study-Act (PDSA) cycle.

Quality improvement initiatives collect data on many measures. Outcome measures reflect what we are trying to achieve, with one outcome measure typically analyzed for a given change idea. The proportion of patients starting dialysis with an eGFR <9.5 mL/min/1.73 m^2^ is an example of an outcome measure. Process measures quantify what we are doing to try to reach a particular outcome.

**Table:** 

***Question 7.*** *Which one of the following is a process measure that we can use to measure the changes we implement in our multidisciplinary kidney clinic?* *a) Proportion of patients with completed symptom management checklist* *b) Proportion of patients starting dialysis with an eGFR >9.5 mL/min/1.73 m^2^* *c) Time (minutes) spent per week calling patients by nursing staff*

*The answer to question 7 is a—the proportion of patients with a completed symptom management checklist. B is an outcome measure, and c is a balancing measure (or measure of trade-offs, or unintended consequences)*

Process measures can be different for each PDSA cycle, with multiple process measures being often evaluated (eg, proportion of patients receiving weekly calls to evaluate symptoms, or the proportion of visits where a symptom management checklist is completed). Last, balancing measures assess unintended consequences or trade-offs. The additional time spent per patient by nursing staff per week or additional time spent calling patients to review symptoms is an example of balancing measures.

**Figure 6.**
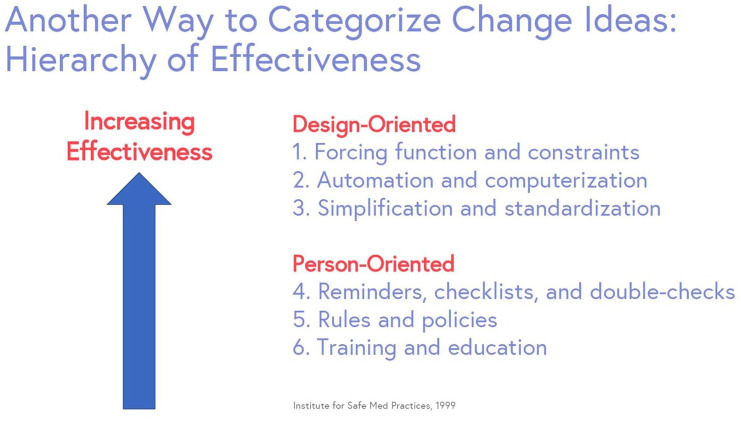
Example of a design and person-oriented change ideas organized by their effectiveness.

**Figure 7.**
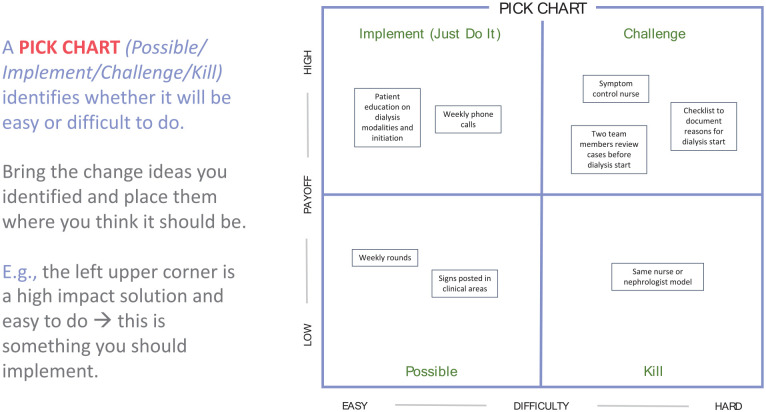
Example of a PICK chart (possible, implement, challenge, kill).

#### Creating a run chart

**Figure 8.**
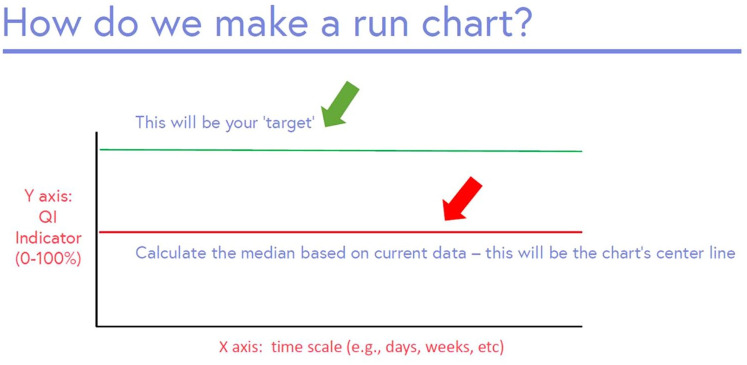
Example of a run chart template.

**Figure 9.**
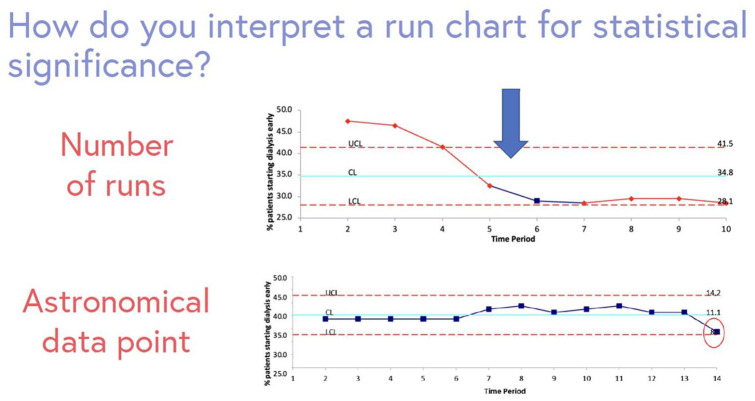
Example of a run chart identifying change in practice over time. Outliers, or astronomical data points are highlighted. A run chart can be interpreted by counting the number of times the line connecting the data points crosses the median which indicates change in practice over time.

**Figure 10.**
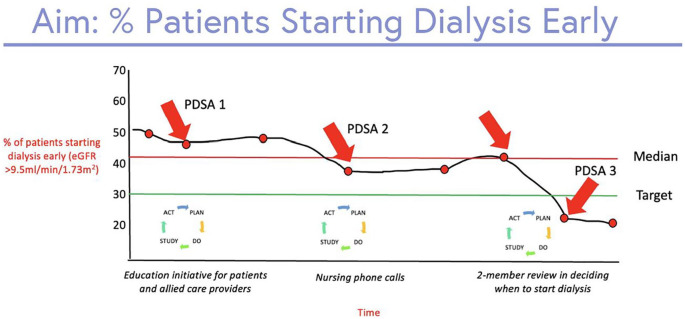
Run chart showing the percentage of patients starting dialysis early (eGFR >9.5 mL/min/1.73 m)^2^. • Arrow 1: No meaningful change with the first PDSA cycle. • Arrow 2: Trend and shift with the second PDSA cycle. • Arrow 3: Upward trend due to lack of regular review of eGFR prior to starting. • Arrow 4: Trend downward with the 3^rd^ PDSA cycle.

A run chart is a graphical display of data, where data from each PDSA can be highlighted (Figure 8).^17^ This visual representation highlights whether the intervention affects the outcome or key process measure, and indicates whether a QI change is showing a nonrandom signal.^17^ The y axis is the QI indicator (eg, 0%-100%), and x axis represents time. Two lines are placed on the chart: (1) median—based on current data, and (2) your chosen target. A run chart highlights if changes lead to improvement, and if that improvement is then sustained. Astronomical data points are points which obviously differ, where all studying the chart would agree the point is unusual.^17^ Figure 9 illustrates a run chart displaying how different PDSA cycles affect the proportion of patients starting dialysis with an eGFR > 9.5 mL/min/1.73 m^2^. A trend represents 5 consecutive points going either up or down (eg, consecutive months where the percentage of patients starting dialysis early decreases), and a shift represents 6 consecutive points that are consistently above or below the median (eg, 6 consecutive months where the percentage of patients starting dialysis early is <35%) (Figure 10).^17^

## Clinical Case—Conclusions

The nursing team starts calling the patient weekly to evaluate for symptoms suggestive of uremia, and a symptom management checklist is introduced. The patient ultimately initiates dialysis at an eGFR of 8 mL/min/1.7 3m^2^ with onset of generalized pruritus and worsening fatigue, in line with current CSN recommendations regarding dialysis initiation.^4^

## Summary

Health care systems have a commitment to continuously improve the quality of care: care that is safe, effective, timely, efficient, patient-centered, and equitable. This nephrology-oriented QI case highlights how a core set of QI principles can be used to develop a QI project. Identifying the quality issue and determining clear goals, engaging the appropriate stakeholders to understand the problem from a range of perspectives, identifying a feasible and measurable change, and using data to track the impact of change on improvement are essential components of all QI initiatives. This case used some of the common methods and principles in quality improvement. This may help spread improvement interventions through growing a culture of improvement and its methodology in renal and the general medical practice. This article will be available in the form of an educational interactive case on the Canadian Society Nephrology (CSN) website at the following address https://bit.ly/41U4wXo. Additional QI-specific tools and resources can also be found at https://www.csnscn.ca/csn-committees/csn-quis-committee/
